# Molecular docking analysis of bioactive compounds from Plectranthus amboinicus with glucokinase

**DOI:** 10.6026/97320630018261

**Published:** 2022-03-31

**Authors:** Umapathy Vidhya Rekha, Rama Varadhachariyar, Ponnulakshmi Rajagopal, Puja Harie Priya, JH Shazia Fathima, Ramajayam Govindan, Chella Perumal Palanisamy, Vishnu Priya Veeraraghavan, Selvaraj Jayaraman

**Affiliations:** 1 Department of Public Health Dentistry, Sree Balaji Dental College and Hospital, Pallikaranai, Chennai-600 100, India; 2 Department of Biochemistry, Sri Venkateshwara Dental College and Hospital, Thalambur, Chennai, India; 3 Central Research Laboratory, Meenakshi Academy of Higher Education and Research (Deemed to be University), Chennai, India; 4 Department of Prosthodontics, Ragas Dental College and Hospital, Uthandi, Chennai, India; 5 Department of Oral and Maxillofacial Pathology, Ragas Dental College and Hospitals, Chennai, India; 6 Multi Disciplinary Research Unit, Madurai Medical College, Tamil Nadu, India; 7 State Key Laboratory of Biobased Materials and Green Papermaking, College of Food Science and Engineering, Qilu University of Technology, Shandong Academy of Science, Jinan 250353, China; 8 Department of Biochemistry, Saveetha Dental College and Hospitals, Saveetha Institute of Medical and Technical Sciences, Saveetha University, Chennai, India

**Keywords:** Diabetes mellitus, Plectranthus amboinicus, Glucokinase, Molecular docking

## Abstract

Natural remedies from medicinal plants are known to be effective and reliable appropriate medicine for illnesses. The current research examined Plectranthus amboinicus' anti diabetic property by docking the bioactive compounds of certain target proteins. We document the molecular docking analysis of bioactive compounds from Plectranthus amboinicus with protein Glucokinase. Molecular docking experiments were carried out in PyRx software. Results of these docking experiments showed that most of the compounds showed very strong interaction with the target protein Glucokinase. Based on the scoring parameters we have selected best four compounds (Rutin, Salvianolic acid, Luteolin and Salvigenin) which showed very good docking score and hydrogen bond interaction for diabetics.

## Background:

Diabetes mellitus is characterized by chronic hyperglycemia and abnormalities in the metabolism of carbohydrates, fat, and protein [1]. Diabetes mellitus has been identified as a major health issue in the world. According to the WHO, about 143 million people worldwide suffer from diabetes [2]. Diabetes is extremely prevalent and severe in developing countries, especially India. India alone has over 40 million diabetics, accounting for approximately 20% of the global diabetes population [3]. In diabetic patients, glucosidase activity is abnormal, which facilitates hypo- or hyperglycemia [4]. Along with insulin, various forms of oral hypoglycaemic agents such as bigunides and sulphonylurea were approved for the management of diabetes. While these medications can help prevent diabetic complications, these can have side effects. The treatment of diabetes mellitus without causing adverse effects continues to be a problem for the medical system. Complementary medicine, on the other hand, has increased in popularity in recent years. As a result, pharmacologists have expressed an interest in developing diabetes treatments based on medicinal plants due to their efficacy, lack of adverse effects in clinical trials, and relative low cost. Numerous indigenous Indian medicinal plants have been described as being beneficial in the effective management of diabetes, and some have been tested [5-6]. The leaf of P. amboinicus is used medicinally for a variety of ailments, most notably coughs, stomachaches, headaches, skin infections, asthma, and urinary disorders [7]. Extracts of this plant have been shown to possess a variety of pharmacological properties, including antioxidant, antibacterial, antimicrobial, anti-inflammatory, and fungi toxic properties [8,9 & 10]. Therefore, it is of interest to document the molecular docking analysis of bioactive compounds from Plectranthus amboinicus with protein Glucokinase.

## Materials and Methods:

### Protein structure preparation:

The crystal structures of Glucokinase (1V4S), a diabetic molecular target, have been downloaded from the PDB database (http://www.rcsb.org). The protein preparation wizard of Argus lab 4.0 (http://www.argus lab.com) was used to optimize and minimize the protein structure. The energies of the protein structure were minimized using the Argus lab Suite's steepest descent minimizes.

### Ligands preparation:

Thirty natural products derived from Plectranthus amboinicus were downloaded from the PubChem database (http://www.pubchem.ncbi.nlm.nih.gov). Then its energy form were minimized and converted to pdbqt format by Open Babel in PyRx 0.8 as ligand for virtual screening. The thirty compounds chosen for this analysis was listed in Table 1(see PDF).

### Virtual screening of compounds from Plectranthus amboinicus:

After optimization, docking against natural compounds from Plectranthus amboinicus was performed using the PDB coordinate data. Auto DockVina was used to perform molecular docking and virtual screening through the PyRX [11,12] interface, providing partial receptor versatility while maintaining high performance and accuracy of results.

### Visualization of docked complexes:

The docked protein- ligand complexes were analyzed and visualized using Pymol molecular visualization software.

## Results and Discussion:

Herbal medicines might continuously consider various biological processes through interactions among multiple compounds and cellular target proteins. As a result, it changes the biological networks from disease to health. Due to the fact that a group of compounds found in the herbal remedy may have a beneficial effect, the dose may be reduced to mitigate toxicity and side effects. The present study screened 30 compounds from Plectranthus amboinicus against the diabetes-specific target protein Glucokinase (1V4S). When compared to the other compounds, the virtual screening revealed that four compounds had the highest inhibitory activity against the target molecule. According to the docking results, Rutin, Salvianolic acid, Luteolin, and Salvigenin have the lowest docked binding energy. The average binding energies varies from- 8.8 and -7.3 kcal/mol. The optimal binding modes for the selected docked complexes were visualized by using the PyMol tool version 1.1 (PyMOL Molecular Graphics System, Version 1.1). The generated images are shown in Figure 1(see PDF), and the associated energy values are described in Table 2(see PDF). Figure 1(see PDF) shows the result of docking analysis of human glukokinase (1V4S) with Rutin it showed the good binding of the protein and ligand with ASP-78,GLY-81,SER-151,LYS-169,THR-228,GLY-229,SER-411,SER-441 and GLU-443 amino acid residues. Compared to other compounds it showed the highest docking score of-8.8kcal/mol. Figure 1b(see PDF) showed the interaction between the glukokinase and Salvianolic acid. It formed nine hydrogen bonds through the amino acids of GLY-81, THR-82, SER-151, LYS-169, THR-228, GLY-229, ASP-409, SER-411 & LYS-414. This also showed very good binding to target protein docking score of -7.6 kcal/mol. Luteolin also showed efficient binding with glukokinase receptor with docking score of -7.4 Kcal/mol. It formed the three H-bond interaction with amino acids SER-151, ASP-205 and THR-228 respectively. All these interaction were shown in Figure 1(see PDF). The compounds Salvigenin formed two H bond interaction (LYS-169 and GLU-443) with glukokinase receptor and also showed the good docking score -7.3 kcal/mol. This was showed in Table 2(see PDF) and can be seen in Figure 1(See PDF). This interaction helped to intercalating the compound in the active site of the Target protein. Glucokinase is required for glucose homeostasis control and is expressed exclusively in liver and pancreatic beta cells. By ensuring a gradient for glucose transport through hepatocytes, regulation of hepatic glucose disposal promotes the glucokinase. Glucokinase is involved in the control of insulin release in beta cells as a result of the cell's glucose supply. In diabetic patients, inadequate or deficient insulin disrupts carbohydrate metabolism, resulting in decreased activity of metabolic enzymes such as glucokinase, resulting in impaired glucose consumption and increased hepatic glucose output. As a result of this, we hypothesized that the selected compounds would increase glucokinase activity, thereby increasing glucose consumption and consequently lowering blood sugar levels.

## Conclusion:

We document the molecular docking analysis of bioactive compounds (Rutin, Salvianolic acid, Luteolin, and Salvigenin) from Plectranthus amboinicus with protein Glucokinase for further consideration in drug discovery.
